# Ecological and behavioural risk factors of scrub typhus in central Vietnam: a case-control study

**DOI:** 10.1186/s40249-021-00893-6

**Published:** 2021-08-19

**Authors:** Hanh Thi Duc Tran, Jan Hattendorf, Hung Manh Do, Thanh Tien Hoang, Hang Thi Hai Hoang, Hoa Ngoc Lam, Mai Kim Huynh, Lan Thi Hoang Vu, Jakob Zinsstag, Daniel Henry Paris, Esther Schelling

**Affiliations:** 1 Department of Epidemiology and Public Health, Swiss Tropical and Public Health Institute, Basel, Switzerland; 2 Department of Epidemiology, Hanoi University of Public Health, Hanoi, Vietnam; 3 University of Basel, Basel, Switzerland; 4 Department for Infectious Disease Control and Prevention, Nha Trang Pasteur Institute, Nha Trang, Khanh Hoa Vietnam; 5 Institute of Gastroenterology and Hepatology, Hanoi, Vietnam; 6 Department of Microbiology and Immunology, Nha Trang Pasteur Institute, Nha Trang, Khanh Hoa Vietnam; 7 Faculty of Medicine, University of Basel, Basel, Switzerland; 8 Department of Medicine, Swiss Tropical and Public Health Institute, Basel, Switzerland; 9 Vétérinaires Sans Frontières Suisse, Bern, Switzerland

**Keywords:** Ecological, Environmental, Behaviour, Risk factor, Scrub typhus, *Orientia tsutsugamushi*, Vietnam

## Abstract

**Background:**

The risk factors for scrub typhus in Vietnam remain unknown. Scrub typhus caused by *Orientia tsutsugamushi* often presents as an undifferentiated febrile illness and remains under appreciated due to the limited availability of diagnostic tests. This tropical rickettsial illness is increasingly recognized as an important cause of non-malaria acute undifferentiated fever in Asia. This study aimed to investigate behavioural and ecological related risk factors of scrub typhus to prevent this potentially life-threatening disease in Vietnam.

**Methods:**

We conducted a clinical hospital-based active surveillance study, and a retrospective residence-enrolment date-age-matched case–control study in Khanh Hoa province, Vietnam, from August 2018 to March 2020. Clinical examinations, polymerase chain reaction and enzyme-linked immunosorbent assay IgM tests were applied to define cases and controls. All enrolled participants filled out a questionnaire including demographic socio-economic status, personal behaviors/protective equipment, habitat connections, land use, and possible exposure to the vector. Multivariable conditional logistic regression was used to define the scrub typhus associated risk factors.

**Results:**

We identified 44 confirmed cases and matched them with 152 controls. Among cases and controls, the largest age group was the 41–50 years old and males accounted for 61.4% and 42.8%, respectively. There were similarities in demographic characteristics between the two groups, with the exception of occupation. Several factors were significantly associated with acquisition of scrub typhus, including sitting/laying directly on household floor [adjusted *OR* (a*OR*) = 4.9, 95% *CI:* 1.6–15.1, *P* = 0.006], household with poor sanitation/conditions (a*OR* = 7.9, 95% *CI:* 1.9–32.9, *P* = 0.005), workplace environment with risk (a*OR* = 3.0, 95% *CI:* 1.2–7.6, *P* = 0.020), always observing mice around home (a*OR* = 3.7, 95% *CI:* 1.4–9.9, *P* = 0.008), and use of personal protective equipment in the field (a*OR* = 0.4, 95% *CI:* 0.1–1.1, *P* = 0.076).

**Conclusions:**

Ecological and household hygiene-related factors were more associated with scrub typhus infection, than individual-level exposure activities in the hyper-endemic area. These findings support local education and allow people to protect themselves from scrub typhus, especially in areas with limitations in diagnostic capacity.

**Graphical abstract:**

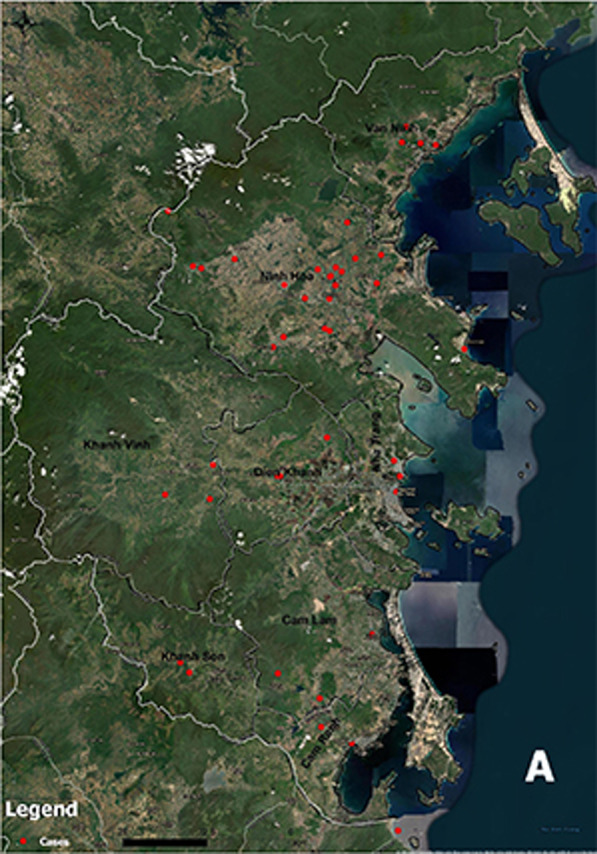

**Supplementary Information:**

The online version contains supplementary material available at 10.1186/s40249-021-00893-6.

## Background

Scrub typhus is a zoonotic infectious disease caused by *Orientia* spp. Humans can be exposed to this bacterium through bites of infected larval-stage trombiculid mites, which are found in rodents of forests and rice fields across the Asia–Pacific region [1–4]. Trombiculid mites have a metamorphosis life-cycle; Female mites lay fertilised eggs in soil, from which 5–7 days later, 6-legged larva (chigger) hatch. These chiggers display host-seeking behaviour by forming clusters on leaves, grasses and twigs above soil surface, and can survive in outdoor environments for weeks-months without a vertebrate host. Chiggers have a large variety of hosts, including maintenance hosts i.e. small mammals (rodents and shrews), ground-dwelling birds, and incidental hosts i.e. larger mammals including humans. Only monkeys, gerbils, hamsters and humans are known to suffer clinically from scrub typhus [1, 5–7]. In severe cases, the disease can progress to multi-organ failure, with pathologic lesions in lungs, kidneys, liver, and brain [8–10]. Absence of eschars, the scrub typhus-specific symptom, makes clinical diagnosis challenging. Currently, standard confirmation tests are for scrub typhus antigen polymerase chain reaction (PCR) and for serologic diagnosis the indirect immunofluorescence assay (IFA) [11]. However, laboratory diagnosis shortly after infection remains difficult, as antibodies do not reach detectable levels for 5–10 days after onset, and the level of *Orientia* bacteria in the blood for PCR, only reaches detectable levels during acute episodes and is inapparent after appropriate initial antibiotic treatment [12, 13]. Therefore, scrub typhus is often misdiagnosed, especially in low-middle income countries with limited laboratory capacity [14].

Scrub typhus is a severe public health problem, with one billion people at risk globally, causes an estimated one million cases every year, and has became a leading cause of treatable non-malarial febrile illness [14, 15]. Scrub typhus is a serious public health problem in the Asia–Pacific [16]. In Vietnam, scrub typhus is re-emerging after 40 years of neglect [17]. Only sporadic cases were registered after 1970. However, in 1995, 45 scrub typhus patients were reported in Quang Ninh province, and 449 patients from 2000 to 2002 [18]. Nadjm et al. [19] reported 255 confirmed cases from 24 northern provinces referred to the National Hospital of Tropical Diseases between 2001 and 2003. Khanh Hoa province confirmed 125 scrub typhus cases per year in 2013/2014, resulting in an estimated incidence of 1.1 per 10 000 [20]. However, laboratory capacity for scrub typhus diagnosis in Vietnam is not yet designated to cope with increasing numbers. Due to late diagnosis and treatment, Bach Mai national hospital estimated a complication rate of 17% and a mortality rate of 1.2% among 251 confirmed patients in 2003 [19, 21].

Epidemiological and ecological information on scrub typhus is very scant in Vietnam. Given the transmission of the bacteria via chiggers, factors at the community level need to be considered to prevent scrub typhus: household sanitation, household/working surroundings, agricultural activities and personal protective measures during outdoor activities [22–24]. As crop fields are an important reservoir for chiggers, farmers are considered a high-risk group for scrub typhus [25]. However, the study in Khanh Hoa in 2013 showed patients with different occupations, including farmers (32%), students (10%), private industry workers (7%), administrative staff (4%), and 1–5% other professions: gardeners, traders, housewives, manual laborers, retirees, tour guides and soldiers [26]. These diverse findings suggest that behavioral risk factors associated with living and working environments in endemic areas are more relevant than occupation. Up to date, no further analyses on behavioral factors were performed in Vietnam.

There is an urgent need to gain a better understanding of disease transmission among humans within their ecosystem to make recommendations on practical preventive measures and fostered case-detection—this study aimed to investigate behavioural and ecological related risk factors of scrub typhus.

## Methods

### Study site

The study took place in Khanh Hoa from August 2018 to March 2020. Khanh Hoa (Latitude 12°N and Longitude 109°E) is in coastal South Central of Vietnam. The population was 1.2 million residents (2019) [27], in 9 districts/townships. The coastal line is 385 km with many lagoons, bays, and islands. Forests and hilly landscapes cover more than half of province. Khanh Hoa is one of the few provinces with a higher gross product from fishing than from agriculture. The tropical savanna climate allows perennial grasses to grow all year, leads to an open shrub layer [28], suitable environment for chigger abundance and scrub typhus transmission.

There are 12 hospitals in Khanh Hoa: 11 public hospitals [Khanh Hoa Provincial Hospital; Ninh Hoa Hospital (Provincial Hospital Branch), Khanh Hoa Hospital for Tropical Diseases; and 8 district hospitals] and Military Hospital 87. Nha Trang Pasteur Institute (IPN) located in Khanh Hoa is one of two Pasteur Institutes in Vietnam, operating directly under authority of the Vietnamese Ministry of Health. Khanh Hoa is known to be endemic for scrub typhus for over 50 years. Scrub typhus was reported among United States Air Force personnel in Khanh Hoa in 1969 [29]. In 2013–2014, 125 confirmed cases per year occurred in 8 of the 9 districts [20].

### Study design

We investigated scrub typhus risk factors using a hospital-based clinical active surveillance, and a retrospective residence-enrolment date-age-matched case-control study to determine risk factors and protective measures associated to disease.

### Hospital-based clinical surveillance

Active surveillance was done at the Military Hospital 87, and 10 of 11 public hospitals of Khanh Hoa, excluding Truong Sa Island district hospital due to accessibility.

All clinical diagnoses during hospitalization admission were made by trained local physicians following suspected acute scrub typhus case definition (Additional file 1: Table S1). All patients satisfying criteria were asked for their informed consent to be enrolled. Demographic and clinical data was collected with standardized questionnaires by trained health staffs. Blood specimens were collected from all enrolled patients at admission and discharge [each 2 tubes, with and without ethylenediaminetetraacetic acid (EDTA)]. Using EDTA blood samples, buffy coat and plasma centrifugation (616 × *g* during 15 min) was conducted within 24 h by trained technicians and stored at 2–8 ℃ in the hospital laboratory of [30], before transfer to IPN, for PCR and enzyme-linked immunosorbent assay (ELISA) [30]. All samples were labeled with the subject’s unique identification number (ID) and preserved in −20 ℃ freezer at the Department of Microbiology and Immunology until further processing.

### Case-control study and sample size

For each confirmed acute scrub typhus case (Additional file 1: Table S1), residence-enrolment date-age-matched controls were enrolled upon their informed consent.

Using Stata software version 14.0 (StataCorp, Texas, USA), we initially calculated a sample size of 128 cases and 512 controls for a matched case-control study, to detect a true odds ratio of 0.5 with 90% power at the 5%-significance level. Taking a ratio of cases and controls of 1:4, a prevalence of exposure (wearing a long-sleeved shirt/trousers for outdoor activities) of 66% among controls and a correlation of exposure between cases and control (rho) of 0.2 were assumed. Facing difficulties with enrolment of cases and controls due to COVID-19 pandemic, we adjusted our calculation and aimed for a more common 80% power. We calculated that 50 cases and 200 controls would be sufficient to detect an odds ratio of 0.4 with 80% power using the same set of assumptions.

For each confirmed case, we enrolled four matched controls: two hospital and two community controls. The inclusion and exclusion criteria of cases and controls were according to the Vietnam national guideline on diagnosis and treatment of infectious diseases 2016 [31] (Additional file 1: Table S1).

Hospital and community controls were defined as residents with no acute scrub typhus, without fever or with fever ≥ 4 days. The 4 day criteria accounted for sufficient time to develop a detectable immunoglobulin M (IgM) titer increase [32]. Based on evidence of macro-level factor effects on scrub typhus exposure risk, i.e. provincial climate (dry/rainy seasons), the controls were enrolled no longer than one month since the date of the case confirmation.

To prevent overmatching, hospital controls were selected in the same district as the case, however from any other commune than the case’s commune. Regarding community controls, for each confirmed case, a list of community controls was prepared by the commune health center, including all eligible persons living around the case household within 500 m, but not next door [7]. Two persons randomly chosen from the list were invited to participate and asked for their informed consent. At time of enrolment, blood samples were taken with the same sampling/storage procedure as described for suspected cases.

Using a residence-enrolment date-age-matched case-control study to determine disease risk factors allowed to minimise effects of ecological confounders related to residence such as mice density, changes of humidity, rain, temperature affecting chigger abundance, its chance to stick on people, and therefore changing exposure probability of people. Enrolment within one month limited changes in households’ (HH) context and conditions. Age-matched case-control design helped to control potential confounders related to age such as typical work activities, occupations, behavior routines among each age group. While persons in the same communes (case-community controls) were likely to share some same working behaviours and personal protective equipment (PPE) wearing routines, the case–control design for both hospital and community controls was thought to address disadvantages of case-community controls while testing risk factors based on varied behaviours and household contexts across different areas.

### Laboratory diagnostic assays

Buffy coat and plasma specimens from cases and controls were tested at IPN, using semi-nested PCR for *Orientia* spp. and using IgM enzyme linked immunosorbent assay (ELISA), (Scrub Typhus Detect™ IgM ELISA, InBios International Inc., Seattle, WA, USA) for detection of antibodies to *O. tsutsugamushi* antigens.

#### PCR assay

A novel in-house semi-nested PCR on buffy coat, developed by IPN, was used for detection of partial 56-kDa outer membrane protein gene of *O. tsutsugamushi* [30]. Primers included 2 forward primers with the sequence of (F1): CAATGTCTGCRTTGTCRTTG; (F2): CCKTTTTCIGCTRGTGCGATAG and one reverse primer with sequence of (R): ATAGYAGGYTGAGGHGGYGTAAG. PCR was done with specimens of 114 suspected cases with peripheral blood mononuclear cells (PBMCs) samples (10 suspected cases without PBMCs).

#### ELISA assays

The Scrub Typhus Detect IgM ELISA (part no. 500242, Lot no. XM5033; InBios International Inc., Seattle, WA, USA) was used for 395 serum samples of 114 suspected cases (95 paired sera hospital admission and discharge, and 19 single admission sera), 103 hospital controls, and 83 community controls. The ELISA used recombinant p56kD type specific antigens of *O. tsutsugamushi* Karp, Kato, Gilliam, and TA716 strains to detect scrub typhus IgM antibodies. The manufacturer’s manual was followed exactly. All sera were tested at a 1:100 dilution and the absorbance measured at 450 nm using a microplate reader to give a final optical density (OD 450 nm) result. The test kit was validated for diagnosis of acute scrub typhus in Asian countries [33, 34]. The OD positivity cut-off titer of 0.8 was used—this had previously shown a sensitivity of 91.5%, specificity of 88.3% for admission samples, and sensitivity of 69.8% and specificity of 89.5% for convalescent samples in another study under comparable conditions [34].

### Building the questionnaire

The individual questionnaire was built on framework of landscape determinants of disease transmission [35]. The same questionnaire was applied to cases and controls. Participants were asked about 5 main items composed of several specific topical questions: (i) socio-economic status, (ii) behaviors related to land/sand/soil/grass/bushes and their PPE, (iii) species’ habitat connections, (vi) land use, and (v) vector contact (Additional file 4: Figure S1).

To develop knowledge around the 5 topics, 12 in-depth interviews with two local scrub typhus epidemiologists, four heads of community health centers (where scrub typhus cases occurred the past years) and six residents (farmers, forest workers, governmental officers) explored local cultivation routines, farming seasons, PPE for daily working use, outdoor activities, and local languages. Following this contextualized “daily-life” assessment, a structured questionnaire and an environment observation checklist were constructed.

### Pre-testing and revision of the questionnaire

The questionnaire was pretested with 30 residents including former scrub typhus patients. The questionnaire was revised according to (i) specificity of questions, (ii) understandability and clearness, (iii) order (reflecting a daily life cycle), (iv) suitability with local context, (v) jumping questions, (vi) language, and (vii) duration of interview. Few cross-check questions were designed information quality checks. Data from pre-testing was not included in our study. The final questionnaire included a total of 96 questions and took about 40 min to complete (30 min for questions and 10 min for the environment observation checklist).

### Data collection

Interviews with cases were conducted within 30 days after laboratory confirmation. In parallel, controls were enrolled, tested and interviewed. Data collectors worked at the Department of Epidemiology, IPN, and the team of four all had experiences from the study in 2014 [36]. A trained laboratory technician joined the field data collection team. Every day, the data supervisor checked total numbers and content of all (paper-based) forms collected. Incomplete forms were completed by the data collector or after re-contacting the participants. This was the case in a total of 10% of questionnaires.

Coordinates of all participants’ households were collected using a short form built in Open Data Kit (ODK) on Android devices (Samsung table) [37], before uploading at http://sg.smap.com.au/. GPS accuracy was set up ± 5 m.

### Data management and quality

The unique ID included a group ID. The composite code was cross checked. The completed questionnaires were double entered by 2 independent data entry clerks, using Epi data 3.1 (EpiData Association, Odense M, Denmark). After that the data was compared using Epi data 3.1 Mismatches were corrected case by case using Stata 15.0 (StataCorp, Texas, USA) to have a clean dataset.

### Statistical analyses

#### Generating new variables

Five PPEs for field work (socks, boots, long/extra shirt, long/extra trousers, and gloves) were in 2 questions: one binary, and one on the frequency of use on a range from 1 to 10 that was further categorized to the binary variable “use of PPE in the field”. “Use of PPE in the field” meant that the person used all 5 PPEs with a minimum frequency of 5/10 times when working in the field.

We used a pre-specified meaningful grouping algorithm to combine related exposure variables into a single binary or ordinal composite variable: (a) “field work group”, (b) “work around house group”, (c) “household (HH) with poor sanitation/conditions”, (d) “HH surroundings with risk” and (e) “workplace environment with risk”. The key risk habitats for the presence of infected chiggers in Southeast Asia were (i) forests, bushes, shifting cultivation area, and (ii) water meadows including grassy edges of water bodies and seepages in drier areas [7, 38–42]. All participants/HH/workplace environment characteristics that related to at least to one of these key risk habitats and were defined as the highest risk group. The others were defined as lower risk groups.

In detail, “field work group” was generated as an ordinal composite variable with 5 sub-groups from highest to lowest risk, including: (i) work in forest/hilly field and others, (ii) work in vegetable garden and others (except forest/hilly areas), (iii) work in sugar cane/crop/rice field and others (except forest/hilly areas/vegetable garden), (iv) work in fruit/industrial tree gardens and others (except forest/hilly areas/vegetable garden/sugarcane/crop/rice field), and (v) no work related to land/sand. By the same way, “work around house group” was created as an ordinal composite variable with 5 sub-groups, containing: (i) watering plants/bonsai or carpenting and others, (ii) cleaning around house and others (except watering plants/bonsai or carpenting), (iii) clearing bushes/barns and others (except watering plants/bonsai, carpenting, cleaning around house), and (iv) no activities around house. “HH with poor sanitation/conditions”, the binary composite variable, was generated as HH with at least one of following characteristics: bushes within 5 m, a mud yard, a mud house floor, or drainage on yard. Two other binary composite variables, “HH surroundings with risk” and “workplace environment with risk” were defined as HH or workplace surrounded by at least one of four natural characteristics: in/close to forest, in/close to hilly field, near water bodies within 100 m or bushes within 10 m.

These composite variables were very useful in examining effect of the full set of related exposure variables, while people or HH could have one or many related exposures of scrub typhus.

### Statistical analyses

We have used composite (as described above) and single variables from the questionnaire. Descriptive statistics included variables to explore major differences between groups. Comparisons of demographic, social, and potential risk factor variables between cases and all controls were analysed using univariable conditional logistic regression. Strength of associated exposures for scrub typhus cases as opposed to controls was expressed by the matched odds ratio. In primary analyses, we pooled both control groups to ensure sufficient statistical power. We used conditional logistic regression to estimate odds ratios and corresponding 95% confidence intervals. In subsequent analyses, we analysed two control groups separately. Community controls were analysed with conditional logistic regression. For hospital controls we preferred adjusted logistic regression as recommended [43]. All analyses were conducted using Stata 15.0 (StataCorp, Texas, USA).

In initial analyses, all potential risk factors were selected by biological plausibility, professional knowledge via literature review, and prior analyses adjusted for field study experience before consideration in models. Subsequently, all potential explanatory variables with *P* < 0.1 in matching univariable models were retained and considered in multivariable models using pooled sample data. The multivariable model was derived using manual backward selection, and considered for effects of retained explanatory variables, confounders and interaction terms. Comparing Akaike information criterion (AIC) of the models, we decided which model had the best fit to our data. In addition, we had the other two models, one comparing cases and hospital controls (subsample 1) and one for cases and community controls (subsample 2). Using the selected potential risk factor variables, the same data analyses procedure described above was applied to two subsamples. We present here results of the final model.

To build distribution maps, satellite imagery of Khanh Hoa captured from Google Earth was used to create a base-map. GIS software ArcGIS 10.6.1 (Esri, California, USA) was used for mapping.

## Results

**Fig. 1:**
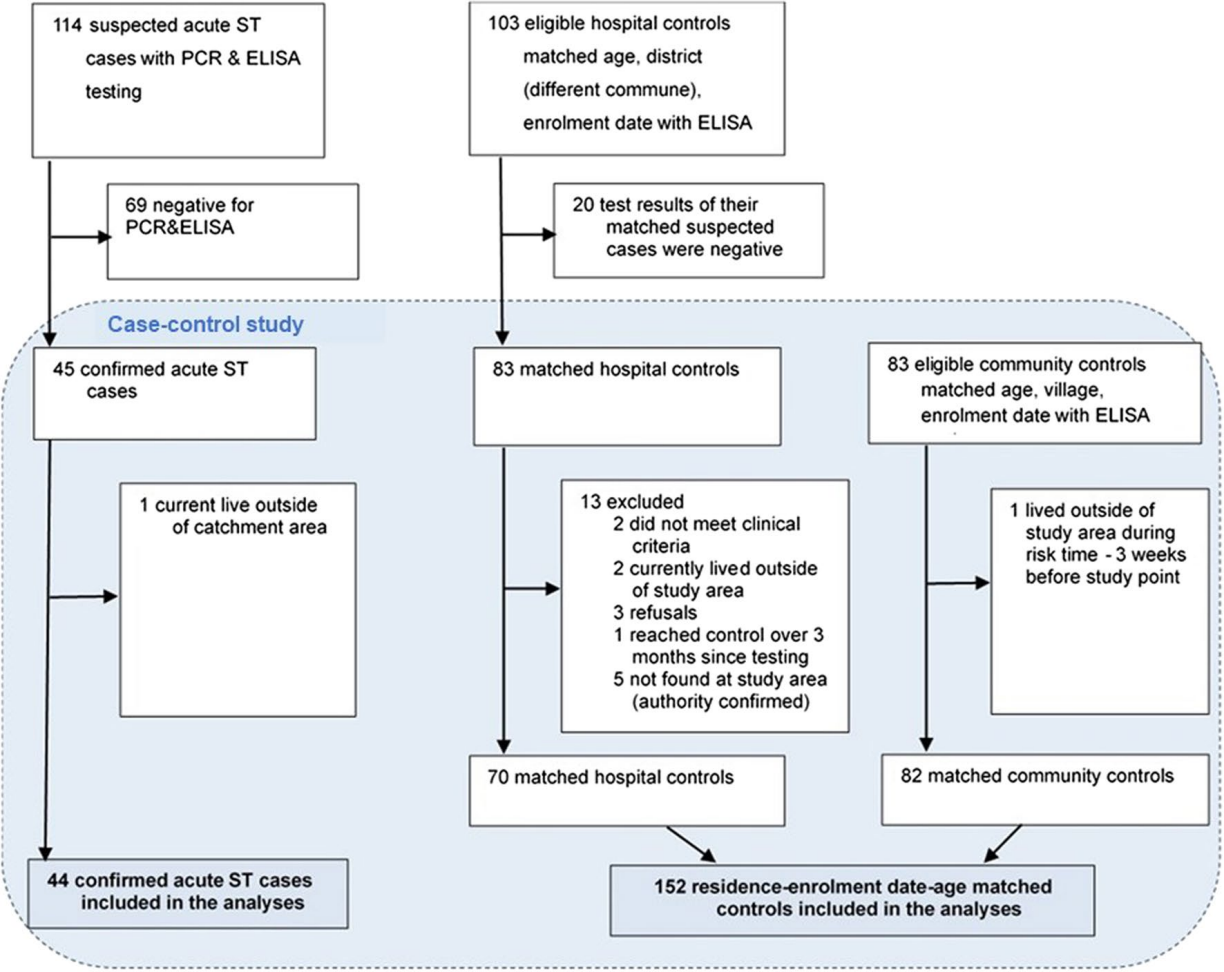
Study flow chart: overview of enrolment of scrub typhus cases, hospital and community controls. ST: scrub typhus; PCR: Polymerase chain reaction; ELISA: Enzyme-linked immunosorbent assay

The study flow chart is shown in Fig. 1. A total of 114 suspected acute scrub typhus cases were initially enrolled to the study. Paired blood specimens (admission and discharge) were collected from 95 suspected cases and admission samples alone from 19 ones. Buffy coats were collected from 104 suspects. Forty-five of the 114 suspects were positive with PCR buffy coat and/or ELISA IgM. After exclusion of one positive due to living outside the study area, we included 44 confirmed cases in the case–control study. Eighty-three hospital controls were initially aligned to the matching-criteria to the 44 confirmed cases, however, 13 of these were excluded for other criteria. Finally, 70 hospital controls were included. We enrolled 82 eligible community controls. In summary, we included data of 196 participants, whereof, 44 confirmed scrub typhus cases and 152 controls.

### Socio-demographic findings and geographic distributions

Among cases and controls 61.4% and 42.8% were males, respectively. The largest age group was 41–50 years old, and comparable between the case and control groups. The two groups were balanced with respect to ethnicity, education level, family size, excepting occupations (9.1% farmers among cases and 21.1% among controls) and working in forests/mountain fields (27.3% among cases and 11.2% among controls). The majority of cases and controls had no history of scrub typhus (90.9% and 98.7%, respectively) (Table 1).

**Table 1:** Socio-demographic comparison between scrub typhus cases and controls, Khanh Hoa, August 2018–March 2020

Variables	Cases (*n* = 44) *n* (%)	Controls (*n* = 152) *n* (%)	*P*-value	m*OR* (95% *CI*)^a^
Age group, years				
≤ 30	12 (27.3%)	45 (29.6%)	–	–
31–40	11 (25.0%)	27 (17.8%)	0.29	1.9 (0.6–6.4)
41–50	13 (29.6%)	47 (30.9%)	0.78	1.2 (0.3–5.3)
≥ 51	8 (18.2%)	33 (21.7%)	0.98	1.0 (0.2–6.5)
Sex				
Male	27 (61.4%)	65 (42.8%)	0.05	2.0 (1.0–4.1)
Female	17 (38.6%)	87 (57.2%)	–	–
Ethical group				
Kinh	38 (86.4%)	125 (82.8%)	0.35	1.8 (0.6–5.9)
Others	6 (13.6%)	26 (17.2%)	–	–
Education				
Illiteracy	2 (4.7%)	10 (6.6%)	–	–
Primary school	16 (37.2%)	35 (23.0%)	0.34	2.3 (0.4–12.4)
Secondary school	11 (25.6%)	60 (39.5%)	0.87	0.9 (0.2–4.7)
High school	11 (25.6%)	30 (19.7%)	0.56	1.6 (0.3–8.7)
University and higher	3 (7.0%)	17 (11.2%)	0.69	0.7 (0.1–5.0)
Occupation				
Not related field/sea/sand	16 (36.4%)	78 (51.3%)	–	–
Farmer	4 (9.1%)	32 (21.1%)	0.17	0.6 (0.4–2.1)
Gardening, growing vegetables	4 (9.1%)	6 (4.0%)	0.11	3.5 (0.8–16.0)
Working in forests/ mountain fields	12 (27.3%)	17 (11.2%)	0.006	**4.5 (1.5–12.9)**
Others	8 (18.8%)	19 (12.5%)	0.24	1.9 (0.7–5.4)
Number of people living in same household				
1–3 people/household	9 (20.5%)	40 (26.5%)	–	–
4–5 people/household	22 (50.0%)	75 (49.7%)	0.43	1.4 (0.6–3.5)
≥ 6 people/household	13 (29.6%)	36 (23.8%)	0.30	1.7 (0.6–4.8)
History of scrub typhus				
Participants 2 years prior to study				
Yes	4 (9.1%)	2 (1.3%)	0.02	7.3 (1.3–39.9)
No	40 (90.9%)	150 (98.7%)	–	–
Family members 2 years prior to study				
Yes	2 (4.7%)	5 (3.3%)	0.64	1.5 (0.3–8.5)
No	41 (95.4%)	147 (96.7%)	–	–
Number of years living in same house				
≤ 1 year	2 (4.6%)	2 (1.3%)	–	–
2–3 years	2 (4.6%)	6 (4.0%)	0.48	0.4 (0.04–4.6)
≥ 4 years	40 (90.9%)	144 (94.7%)	0.22	0.3 (0.04–2.1)

^a^m*OR*: matched odds ratio, using conditional logistic regression **bold:* P*-value < 0.05

**Fig. 2:**
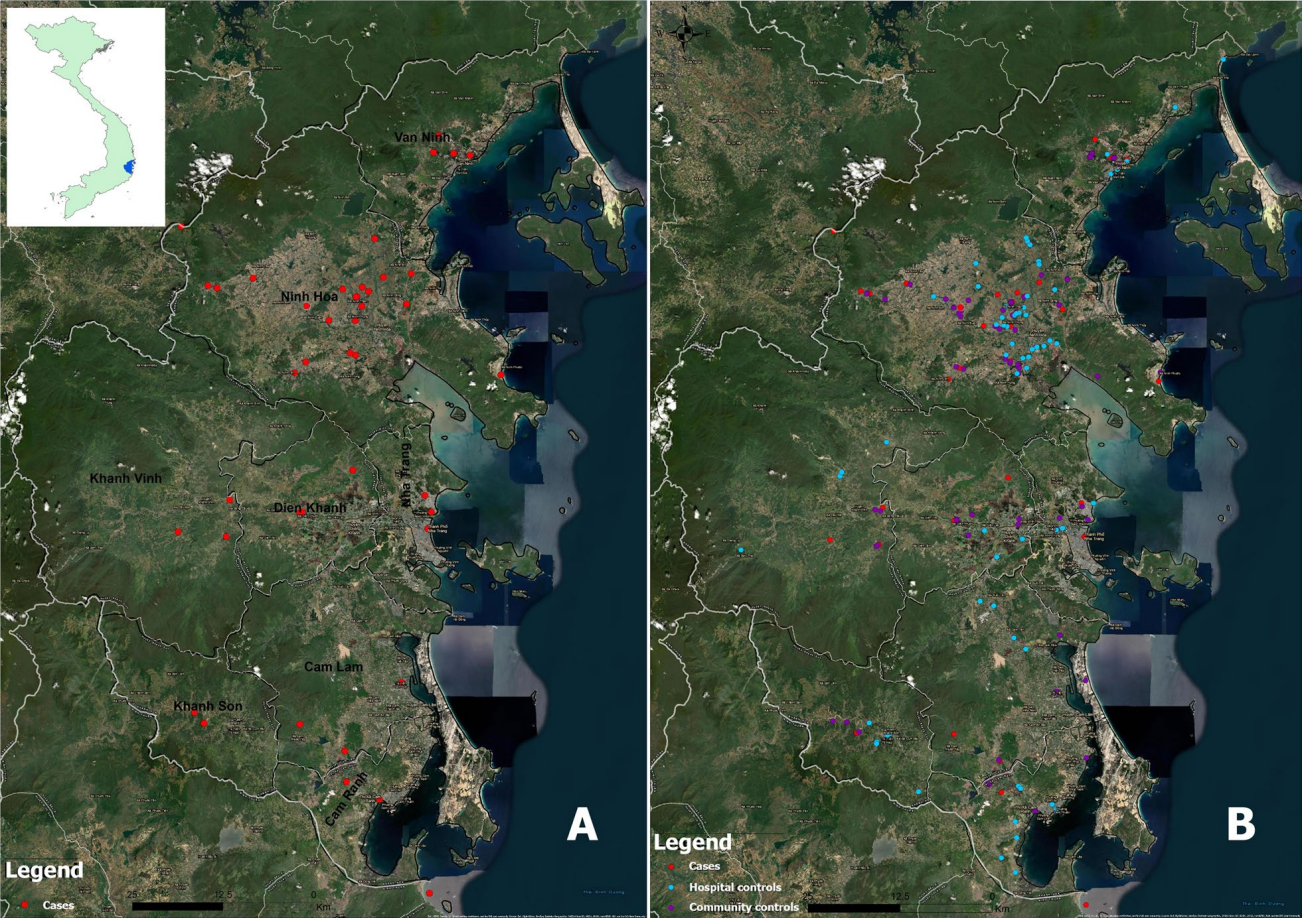
Geographic distributions of scrub typhus cases and controls, Khanh Hoa, August 2018–March 2020

We plotted geographic distributions of scrub typhus confirmed cases (A) and controls (B) (Fig. 2). Scrub typhus cases occurred in all 8 districts. There were differences in numbers of confirmed cases across the districts. Most cases occurred in Ninh Hoa while a few were in other districts. Cases were observed in all zones, including flat areas, near mountains, near forests, or alongside beaches.

### Risk factors

Exposures associated with scrub typhus in univariable conditional logistic regression are provided in Table 2. Cases were more likely than controls to get scrub typhus when they worked in forest/hilly areas [matched odds ratio (m*OR)* = 23.9, 95% *CI:* 5.8–98.2], worked in fruit/industrial tree gardens (m*OR* = 10.5, 95% *CI:* 1.6–69.4), urinated in the forest/near bushes/field (m*OR* = 5.6, 95% *CI:* 2.4–13.1), passed regularly at the river side (m*OR* = 2.8, 95% *CI:* 1.4–5.6), used the same work clothes the next day (m*OR* = 2.4, 95% *CI:* 1.0–5.6), sat/laid directly on the HH floor (m*OR* = 3.9, 95% *CI:* 1.7–8.7).

**Table 2:** Exposures associated with scrub typhus in Khanh Hoa, Vietnam, August 2018–March 2020

Variables	Cases (*n* = 44) *n* (%)	Controls (*n* = 152) *n* (%)	*P*-value	m*OR* (95% *CI*)^a^
Field work group				
Work in forest/ hilly field and others	26 (59.1%)	24 (15.8%)	< 0.001	**23.9 (5.8–98.2)**
Work in vegetable garden and others	6 (13.6%)	15 (9.9%)	0.07	**3.9 (0.9–16.6)**
Work in sugarcane/crop/rice field and others	3 (6.8%)	37 (24.3%)	0.88	0.9 (0.1–5.2)
Work in fruit/industrial tree garden and others	3 (6.8%)	8 (5.3%)	0.02	**10.5 (1.6–69.4)**
No work related to land/sand	6 (13.6%)	68 (44.7%)	–	–
Use of personal protective equipment in the field				
Yes	14 (31.8%)	86 (56.6%)	0.004	**0.3 (0.1–0.7)**
No	30 (68.2%)	66 (43.4%)	–	–
Urinating in the forest/near bushes/field				
Yes	25 (56.8%)	34 (22.4%)	< 0.001	**5.6 (2.4–13.1)**
No	19 (43.2%)	118 (77.6%)	–	–
Using the same work clothes the next day				
Yes	15 (34.1%)	30 (19.7%)	0.05	2.4 (1.0–5.6)
No	29 (65.9%)	122 (80.3%)	–	–
Changing clothes when at home				
Yes	34 (77.3%)	140 (92.1%)	0.01	**0.3 (0.1–0.8)**
No	10 (22.7%)	12 (2.9%)	–	–
Always observing mice around home				
Yes	21 (47.73%)	37 (24.34%)	0.003	**3.1 (1.5–6.6)**
No	23 (52.27%)	115 (75.66%)	.	.
Work around house group				
Watering plants/bonsai or carpenting + others	19 (43.2%)	54 (35.5%)	0.48	1.3 (0.6–2.9)
Cleaning around the house + others	6 (13.6%)	34 (22.4%)	0.46	0.7 (0.2–1.9)
Clearing bushes/barns + others	4 (9.1%)	9 (5.9%)	0.36	1.9 (0.4–7.2)
No activities around house	15 (34.1%)	55 (36.2%)	–	–
Passing regularly at the riverside				
Yes	23 (52.3%)	42 (27.6%)	0.004	**2.8 (1.3–5.6)**
No	21 (47.7%)	110 (72.4)		
Sitting/laying directly on household floor				
Yes	23 (52.3%)	40 (26.7%)	0.001	**3.9 (1.7–8.7)**
No	21 (47.7%)	110 (73.3%)		
Household with poor sanitation/conditions				
Yes	41 (93.2%)	96 (63.2%)	< 0.001	**8.4 (2.4–29.1)**
No	3 (6.8%)	56 (36.8%)		
Household surroundings with risk				
Yes	22 (50%)	45 (29.6%)	0.007	**3.1 (1.4–7.2)**
No	22 (50%)	107 (70.4%)		
Workplace environment with risk				
Yes	30 (68.18%)	58 (38.16%)	0.001	**3.6 (1.7–7.7)**
No	14 (31.82%)	94 (61.84%)	.	.

^a^m*OR*, matched odds ratio, using univariable conditional logistic regression, **bold: *P*-value < 0.05

Always observing mice around home (m*OR* = 3.1, 95% *CI:* 1.5–6.6), HH surroundings with risk (m*OR* = 8.4; 95% *CI:* 2.4–29.1), and workplace environment with risk (m*OR* = 3.6, 95% *CI:* 1.7–7.7) were associated with increased acquisition risk. Lower risks for acquiring scrub typhus were the use of PPE in the field and changing clothes when at home (m*OR* = 0.3, 95% *CI:* 0.2–0.7; and m*OR* = 0.3, 95% *CI:* 0.1– 0.8, respectively).

The most relevant factors associated with scrub typhus in the multivariable conditional logistic regression model are presented in Table 3. We found four significant risk factors and one protective determinant for scrub typhus, including: sitting/laying directly on HH floor [adjusted odds ratio (a*OR*) = 4.9, 95% *CI:* 1.6–15.1], HH with poor sanitation/conditions (a*OR* = 7.9, 95% *CI:* 1.9–32.9), workplace environment with risk (a*OR* = 3.0, 95% *CI:* 1.2–7.6), always observing mice around home (a*OR* = 3.7, 95% *CI:* 1.4–9.9) and, as protective factor with low statistical evidence, use of PPE in the field (a*OR* = 0.4, 95% *CI:* 0.1–1.1).

**Table 3:** Risk factors of scrub typhus resulting from the conditional multivariable logistic regression

Risk factors	Cases (*n* = 44)	Controls (*n* = 152)	m*OR* (95% *CI*)^a^	a*OR* (95% *CI*)^b^	*P*-value
	*n* (%)	*n* (%)			
Sitting/laying directly on household floor	23 (52.3%)	40 (26.7%)	3.9 (1.7–8.7)0.328779	4.9 (1.6–15.1)	0.006
Use of personal protective equipment in the field	14 (31.8%)	86 (56.6%)	0.3 (0.2–0.7)	0.4 (0.1–1.1)	0.076
Household with poor sanitation/conditions	41 (93.2%)	96 (63.2%)	8.4 (2.4–29.1)	7.9 (1.9–32.9)	0.005
Workplace environment with risk	30 (68.18%)	58 (38.16%)	3.6 (1.7–7.7)	3.0 (1.2–7.6)	0.020
Always observing mice around home	21 (47.73%)	37 (24.34%)	3.1 (1.5–6.6)	3.7 (1.4–9.9)	0.008
Sex (males)	27 (61.4%)	65 (42.8%)	2.0 (1.0–4.1)	1.4 (0.5–3.8)	0.518

The model adjusted for: sex, field work group, use of personal protective equipment in the field, urinating in the forest/near bushes/field, using the same work clothes the next day, changing clothes when at home, always observing of mice around home, raising cattle, seeing chickens that you raise have mites, passing riverside, sitting/laying directly on household floor, household with poor sanitation/conditions, household surroundings with risk, workplace environment with risk

^a^m*OR*, matching odds ratio, using univariable conditional logistic regression; ^b^a*OR*, adjusted odds ratio, using multivariable conditional logistic regression

Risk factors among cases-hospital controls (i.e. living in different communes) were not similar to those among cases-community controls (i.e. living in same commune). Among people living in different communes, always observing mice around home, workplace environment with risk were associated with increased scrub typhus risk (a*OR* = 5.4, 95% *CI:* 1.7–17.1; and a*OR* = 4.9, 95% *CI:* 1.6–15.3, respectively). Changing clothes when at home was likely to protect from scrub typhus (a*OR* = 0.1, 95% *CI:* 0.02–0.6). Among persons in same commune, sitting/laying directly on HH floor and adult men were risk factors of scrub typhus (a*OR* = 35.3, 95% *CI:* 3.4–368.8; and a*OR* = 6.3, 95% *CI:* 1.1–34.4, respectively). Use of PPE in the field was a potential factor to protect from bites of mites in same endemic communes (a*OR* = 0.21, 95% *CI:* 0.04–1.09) (Additional files 2, 3: Tables S2 and S3).

## Discussion

Scrub typhus is acquired through the bite of infected chiggers. Based on biological plausibility pathways, we present the discussion in order from proximal (direct chigger exposure) to distal risk factors (always observing mice around home). The five main factors for scrub typhus acquisition in this study were (i) sitting/laying directly on HH floor, (ii) use of PPE in the field, (iii) HH with poor sanitation/conditions, (iv) workplace environment with risk, and (v) always observing mice around home.

### Sitting/laying directly on HH floor

Sitting/laying directly on HH floor was the most direct exposure to acquire scrub typhus in our endemic area (a*OR* = 4.9, 95% *CI:* 1.6–15.1). One likely explanation is that people were bitten by infected chiggers when sitting/laying directly on HH floor. Chiggers of mites of *Leptotrombidium deliense* and *Ascoschoengastia (Laurentella) indica* (species of Trombiculidae mite family) were key chigger species transmitting *Orientia* spp. in Southeast Asia, and found on indoor and outdoor mice [1–3]. In Khanh Hoa, *L. deliense* and *A. indica* were found on house mice, accounting for 11.9% of all detected transmission mites [53]. Among house rodents, the proportion of mite infestation was 82.7% for *Rattus norvegicus*, followed by *R. flavipestus* (66,7%), and *R. exulans* (34,1%). Proportions of *R. norvegicus* and *R. flavipestus* with *Orientia* spp. positivity were 1.7% and 0.24%, respectively (Khanh Hoa, 2013–2014) [44]. One scrub typhus paediatric patient (10 months) in Khanh Hoa was confirmed by IPN in 2013 [45]. Evidence suggests that sitting/laying directly on HH floor, is strongly associated with an increased risk of acquiring scrub typhus.

### Use of PPE in the field

Benefits of PPE usage in preventing scrub typhus were reported. Most authors considered a benefit of wearing gumboots, aprons, long-sleeved shirt, long-sleeved clothes as separate items [45–48]. In this study, we did not observe benefits of single measures—our findings rather suggest that an advantage lies in using the full PPE, including all 5 items: socks, boots, long/extra shirt, long/extra trousers, and gloves, potential to protect persons from bites of mites in the endemic area (a*OR* = 0.4, 95% *CI:* 0.1–1.1). This highlights that people at risk need a full protective equipment to prevent infected mites climbing up any body parts while working/sitting in the field.

### Households with poor sanitation/conditions

Households with poor sanitation/conditions (HH with drainage on ground—not including grassy edges of water bodies, or HH with a muddy/sandy yard or HH with a muddy floor) represented an important risk factor in this study. Water drainage or seepage in drier areas was defined as a crucial micro-ecology favoring the presence of chiggers and increased risk of acquiring scrub typhus [7, 41, 42]. Common chigger habitats are grassy edges, soil dampness, and a well nearby the household [41, 42, 49], but water drainage on the household ground/premises was not considered [20, 50, 51]. A sandy/muddy yard or a muddy house floor take up dampness through water drainage after rainfall, which fosters survival of chiggers. Chigger abundance could be maintained by sprinkling the ground with water after rains in Malaysia [52].

### Workplace environment with risk

Workplace environment with risk (i.e. close to forest/hilly field/water bodies within 100 m or bushes within 10 m) was associated with scrub typhus acquisition (a*OR* = 3.0, 95% *CI:* 1.2–7.6). To note is that more importantly than occupation. Working in forests/hilly fields were common risk factors [24, 45, 53]. However, in previous studies, daily activities were often indicated as individual “long-term”’ activities [53], rather than “workplace environment”. In our study, we expressed “workplace environment with risk” based on the wide diversity of work activities in this surrounding and by duration (about 1 h/day), rather than by one long-lasting single work. In Khanh Hoa, not being named by any specified job title, working close to forests/hilly fields included varied typical occupations (self-business/hired) such as herding cows, mowing grass for livestock, picking up firewood, planting vegetation into forest streams, wood truck driver, loading fruits/sugarcane. Therefore, “workplace environment with risk” was a more relevant and broader definition than a single type of occupation. This approach may be more useful for medical staffs to locate risk areas and initiate preventive measures, than focusing only on specific jobs/occupations.

Working near water bodies (lakes/ponds/streams/channels/wells/irrigation systems surrounded by vegetation or seepages within 100 m) was a major risk factor for scrub typhus [41, 42, 49]. We identified typical local seepages such as sand fields, beaches, coastlines as a special working habitat of people planting garlic on sand, fishermen, throwing fishing nets, and catching seafood. This is geographically relevant for Khanh Hoa [27]. Sandy fields and beaches represent preferred environments for mites and chiggers [7]. *Orientia* spp. vectors and hosts have been described along sandy beaches in Malaysia [54]. *Leptotrombidium arenicola* spp. has been detected in vegetation alongside beaches in Southeast Asia [39, 55].

### Farmer or agriculture work is a “vague” risk factor definition in Vietnam

Farmers represent a high risk population for scrub typhus due to their long time exposure in fields [45, 53, 56]. However, we could not find this, which could be explained by following reasons: (i) different rates of pre-existing immunity through repeated exposure in highly endemic areas; (ii) a patchy distribution of chiggers in the environment (chigger islands), which are dependent on rodent density in this region, (iii) a different perception/definition of occupation as “farmer”, and (iv) multiple people perform multiple work-related activities, representing a “mixed” risk profile.

Association between farmer/occupation and scrub typhus varied across endemic areas, depending on both activity and environment in which cases were exposed to. A hospital-based study reported that agricultural labour was associated with an increased risk for scrub typhus in Jiangsu province, China (a*OR* = 2.9, 95% *CI:* 1.5–5.8) [57]. However, in Uttarakhand, India, housewives (52%) and students (28%) were the two major occupational subgroups among scrub typhus patients (while farmers were 11.11%), thus likely representing previously non-exposed people with no pre-existing immunity, that participated in local harvests in fields, rather than their usual urban-based occupations [58]. Among community-based studies, an association between farming and occurrence of scrub typhus was reported for India (a*OR* = 2.0, 95%*CI:* 1.1–3.5 [24], m*OR* = 10.0; 95% *CI:* 2.7–63.0 [45]) and for the Lao PDR (m*OR* = 2.1, 95% *CI:* 1.0–4.2) [59]. However, in a scrub typhus outbreak in Guangzhou, China (2012), no patients engaged in agricultural activities. The outbreak was finally assigned due to outdoor exercises in a local park [60].

This study revealed that the term “farmer” or “agricultural work” is often perceived as a “vague” definition in Vietnam. This was more pronounced in rural study areas, and often people defined themselves as farmers when their family owned a field, even if they hired others for farming or did no agricultural work for a long time. In-depth clarification of our study revealed that a proportion of 25.5% agricultural labours did no farming within the preceding 3 weeks prior to onset. In contrast, 27.5% persons indicating that they were officers/factory workers/students/small businessmen, involved agricultural/forest activities during that period. Further, a person–hired for agricultural work (cultivating, harvesting for 2–10 days), described themselves as “work-for-hire”, not as “farmer”. Therefore, using occupation/farmer to examine risk of scrub typhus in Vietnam could introduce bias into associations.

People with multiple occupations/work activities were common in our study setting. A participant could have multiple jobs, i.e. a nurse after night work-shift had a day-off and mowed grass for cows; or a hotel waiter worked as gardener for the hotel and as tour guide. Over 50% participants had at least 3 varied activities in the preceding 3 weeks exposure period, often including farming, cultivating crops (hired), cow herding (hired), fishing, planting acacia trees on hilly areas. A high mobility was noted among these patients. In the 3 weeks before disease onset, next to cultivating, a person could work as wood/sugarcane truck driver, loading fruits, herding cows, mowing grass, laying bricks (hired) or coastal fishing. Farming is a seasonal occupation, whereby most do not work full-time in the fields, even during the 3–4 months farming season. Therefore, in “non-farming time”, these people are occupied with other exposures/jobs/activities.

The collated data suggests that farmer/occupation is likely not a clear indicator of scrub typhus infection. Workplace environment is more practical than farmer/occupation to examine that association. Workplace environment, covering and representative for all activities in such risk area, is likely a better approach for a risk assessment of acquiring scrub typhus.

### Always observing mice around home

Always observing mice around home” was one of the risk factors for scrub typhus in this study, similar to other studies [45, 60] (a*OR* = 3.7, 95% *CI:* 1.4–9.9). This finding is supported by available evidence on the presence of *Orientia* spp. infected *Leptotrombidium deliense* spp. and *Ascoschoengastia indica* spp. among house rodents in Khanh Hoa [53].

### Difference in risk factors between hospital and community controls

In independent analyses of case-community controls, we found that sitting/laying directly on HH floor was a risk factor of scrub typhus only among persons in the same commune, whereas “always observing mice around home” was not (a*OR* = 2.7, 95% *CI:* 0.7–10.3, Additional file 2: Table S2). These findings could be explained due to the fact that confirmed cases and their community controls lived in the same area (within 500 m) and shared similar ecological characteristics such as mice/rodent density [7]. Therefore, we did not observe a difference in presence of mice around their houses. However, sitting/laying directly on HH floor was the most important risk factor among persons in the same communes. It was likely the strong evidence on association between direct infected mite exposure and scrub typhus, confirming transmission of *O. tsutsugamushi* spp. among mites and between mites-house mice in these hyper endemic communes. Independent analyses of case-hospital controls (i.e. living in different communes) showed that always observing mice around home was more clearly associated with *O. tsutsugamushi* infection, with a*OR* = 5.4, 95% *CI:* 1.7–17.1 (Additional file 3: Table S3). This finding was also supported for transmission of *O. tsutsugamushi* spp. among mites and house mice in cases’ communes.

### Study highlights

Reported common risk factors for scrub typhus are bushes around house/working place [45, 51, 53, 61–64], always observing mice around home [45, 60], and working near forest/hilly fields [24, 45, 53]. In this study, we highlighted additional factors for Khanh Hoa, including (i) sitting/laying directly on HH floor; (ii) use of full PPE set (socks, boots, long/extra shirt, long/extra trousers, gloves) in the field, (iii) changing of clothes when at home; (iv) HH with sandy or muddy grounds/a muddy floor, or HH with drainage on ground; and (v) workplace near sandy fields. This study assessed ecological and behavioural risk factors, based on the full landscape framework [35] and an ecological epidemiology approach in a comprehensive in-depth questionnaire, which allowed the identification of proximal and more distal ecological factors.

### Consequences for control and elimination

Facing challenges to detect scrub typhus cases in Vietnam despite limitations in diagnostics, the risk factors found in this study, combined with better known clinical symptoms and epidemiology, should be presented in training courses at hospitals and for doctors trained on the emerging scrub typhus. Epidemiological risk factors and illness case-detection have improved with this study.

The factors identified in this study are useful to support establishment of preventive measures, inform regional surveillance, and promote much-needed effective public health responses against scrub typhus after many decades of neglect in Vietnam. Currently, there are no disease prevention and control strategy for scrub typhus or rodents in Khanh Hoa and Vietnam—however, we found this an important risk factor. To note is that rodent control in settlements can also prevent other diseases such as plague or leptospirosis [65]. Facing current limitations in diagnostic capacity for scrub typhus in Central Vietnam, especially at primary health care levels, these findings will support local education and allow local people in hyper-endemic areas to increase their risk factor knowledge and protect themselves from scrub typhus.

## Limitations of the study

This was an exploratory study given that no known set of risk factors for Vietnam could be confirmed. A retrospective study design is prone to recall bias. However, in this study, defining and enrolling participants to the study was no longer than 1 month since the date of the case confirmation and asking for a 3 week exposure period to minimise recall bias. The initial sample size was adjusted due to the COVID-19 pandemic, which reduces statistical power to detect associations between use of PPE in the field and scrub typhus. However, the study was obviously sufficiently powered to detect several important risk factors. Further, our results might only apply for provinces in same ecological and endemic zone like Khanh Hoa, which are the 11 central provinces in Vietnam.

## Conclusions

Ecological and household hygiene related factors such as HH with poor sanitation/conditions, always observing mice around home, and workplace environment with risk were associated with *Orientia* spp. infection, rather than individual-level exposure activities. Use of PPE in the field and changing clothes when at home were potential protective factors.

## Supplementary Information

**Additional file 1: Table S1.** Inclusion and exclusion criteria of scrub typhus suspected, confirmed cases and hospital controls, community controls.

**Additional file 2: Table S2.** Risk factors of scrub typhus resulting from case-hospital control analyses.

**Additional file 3: Table S3.** Risk factors of scrub typhus resulting from case-community control analyses.

**Additional file 4: Figure S1.** Visualisation of 5 main items regarding ST risk factors to be assessed in Khanh Hoa.

## Data Availability

The datasets used and/or analysed during the current study are available from the corresponding author on reasonable request.

## Abbreviations

PCR: Polymerase chain reaction
IFA: Indirect immunofluorescence assay
EDTA: Ethylenediaminetetraacetic acid
ELISA: Enzyme-linked immunosorbent assay
COVID-19: Coronavirus disease 2019
IgM: Immunoglobulin M
PPE: Personal protective equipment
ID: Identification number
HH: Household
AIC: Akaike information criterion.
IPN: Nha Trang Pasteur Institute
