# Supplementary material for: Ecological and behavioural risk factors of scrub typhus in central Vietnam: a case-control study
Source: Infect Dis Poverty. 2021 Aug 19;10:110. doi: 10.1186/s40249-021-00893-6 (PMC8374119; doi:10.1186/s40249-021-00893-6)
Supplement: Supplementary file 1 — Additional file 1: Table S1. Inclusion and exclusion criteria of scrub typhus suspected, confirmed cases and hospital controls, community controls. [file 40249_2021_893_MOESM1_ESM.docx]

Table S1 Inclusion and exclusion criteria of scrub typhus suspected, confirmed cases and hospital controls, community controls

| **Suspected acute scrub typhus case** | **Confirmed acute scrub typhus cases** | **Hospital controls** | **Community controls** |
| --- | --- | --- | --- |
| **Demography** |  |  |  |
| **Inclusion criteria:**   - Age ≥ 16 years old - Resident of Khanh Hoa province - Living in Khanh Hoa province for ≥ 6 months prior to study enrolment | **Inclusion criteria:**  Same as suspects | **Inclusion criteria:**   - Age ≥ 16 years old - Resident of Khanh Hoa province - Living in Khanh Hoa province for ≥ 6 months in the same district and, but different commune as the case, matching for urban – rural areas (according to classification of the statistical office) - +/- 10 years of the case - **Exclusion criteria:** - Army members, tourists | **Inclusion criteria:**   - Age ≥ 16 years old - Resident of Khanh Hoa province - Living in Khanh Hoa province for ≥ 6 months in the same village as the case - Present in the commune 3 weeks prior to enrolment - +/- 10 years of the case   **Exclusion criteria:**   - Army members, tourists |
| **Clinical** |  |  |  |
| **Inclusion criteria**   - Patient with undifferentiated acute fever (temperature using axilla ≥37.5^O^C) and having had at least one of the following twelve secondary symptoms: eschar, non-specific skin rash, headache, myalgia, retro-orbital pain, congestion of the conjunctival blood vessels, tinnitus, lymphadenopathy (regional/body), hepatomegaly, splenomegaly, dry cough, dyspnoea without upper respiratory tract discharge.   **Exclusion criteria:**   - Patients diagnosed with malaria, dengue fever, measles, influenza, bacterial pneumonia, urinary tract infections, based on strong clinical suspicion | **Inclusion criteria**  Same as suspects | **Inclusion criteria:**   - Hospitalized >24 hours - Diagnosed with any disease other than scrub typhus without fever or with ≥ 4 days of fever (1) - Hospitalized within 14 days since the case’s enrolment date.   **Exclusion criteria:**   - Patients with dengue fever, malaria, HIV/AIDS and TB, - Patients with limited mobility caused by past surgeries or illness | **Inclusion criteria:**   - People with without fever or with ≥ 4 days of fever, but without scrub typhus diagnosis   **Exclusion criteria:**   - People with dengue fever or malaria at the time of the study, HIV/AIDS and TB, - Patients with limited mobility caused by past surgeries or illness - People hospitalized within 1 month prior to study enrolment |
| **Laboratory** |  |  |  |
| **Exclusion criteria:**   - Positive (rapid) laboratory test results with malaria, dengue fever (confirmed by NS1), measles, influenza, bacterial pneumonia, urinary tract infections. | **Inclusion criteria:**   - Positive of PCR on buffy coat and/or ELISA IgM with OD cut-off of 0.8 (2) | **Inclusion criteria:**   - The ELISA IgM negative for scrub typhus with OD cut-off of 0.8 (2) | **Inclusion criteria:**   - The ELISA IgM negative for scrub typhus with OD cut-off of 0.8 (2) |
| **Exposure period** |  |  |  |
|  | 21 days (3 weeks) before symptoms/fever onset (1) | 21 days before fever onset or 21 days before hospitalisation if controls have no fever (1) | 21 days before fever onset or before study enrolment (1) |

1. Paris DH, Richards AL, Day NPJ. Chapter 112 - Orientia In: Sussman M, Liu D, Poxton I, Schwartzman J, editors. Molecular Medical Microbiology (Second Edition). Boston: Academic Press; 2015. p. 2057-96.

2. Blacksell SD, Kingston HWF, Tanganuchitcharnchai A, Phanichkrivalkosil M, Hossain M, Hossain A, et al. Diagnostic Accuracy of the InBios Scrub Typhus Detect™ ELISA for the Detection of IgM Antibodies in Chittagong, Bangladesh. Trop Med Infect Dis. 2018;3(3):95.
