# Supplementary material for: Ecological and behavioural risk factors of scrub typhus in central Vietnam: a case-control study
Source: Infect Dis Poverty. 2021 Aug 19;10:110. doi: 10.1186/s40249-021-00893-6 (PMC8374119; doi:10.1186/s40249-021-00893-6)
Supplement: Supplementary file 2 — Additional file 2: Table S2. Risk factors of scrub typhus resulting from case-hospital control analyses. [file 40249_2021_893_MOESM2_ESM.docx]

Table S2 Risk factors of scrub typhus resulting from case-hospital control analyses

| **Variables** | **Cases (N=44)** | **Controls (N=70)** | **aOR (95%CI)^#^** | **P-value** |
| --- | --- | --- | --- | --- |
|  | **n (%)** | **n (%)** |  |  |
| **Gender (male)** | 27 (61.4%) | 35 (50.0%) | 0.9 (0.3-3.0) | 0.890 |
| **Urinating in the forest/near bushes/field** | 25 (56.8%) | 12 (17.1%) | 2.3 (0.7-7.2) | 0.151 |
| **Changing clothes when at home** | 34 (77.3%) | 68 (97.1%) | 0.1 (0.0-0.6) | 0.014 |
| **Always observing mice around home** | 21 (47.7%) | 15 (21.4%) | 5.4 (1.7-17.1) | 0.004 |
| **Household with poor sanitation/conditions** | 41 (93.2%) | 37 (52.9%) | 7.1 (1.6-32.1) | 0.011 |
| **Workplace environment with risk** | 30 (68.2%) | 16 (22.9%) | 4.9 (1.6-15.3) | 0.006 |
| **Age group** |  |  |  |  |
| ≤30 | 12 (27.3%) | 22 (31.4%) | - |  |
| 31-45 | 15 (34.1.0%) | 23 (32.9%) | 0.9 (0.3-3.4) | 0.919 |
| ≥46 | 17(38.6%) | 25 (35.7%) | 1.0 (0.3-3.6) | 0.977 |
| **District with mainly flat area** |  |  |  |  |
| Yes | 25 (56.8%) | 39 (55.7%) | 1.7 (0.5-5.2) | 0.373 |
| No | 19 (43.2%) | 31 (44.3%) | - |  |
| ** The model adjusted for: sex, field work group, use of personal protective equipment in the field, urinating in the forest/near bushes/field, using the same work clothes the next day, changing clothes when at home, always observing mice around home, raising cattle, seeing chickens that you raise have mites, passing riverside, sitting/laying directly on household floor, household with poor sanitation/conditions, household surroundings with risk, workplace environment with risk.* | | | | |
| *^#^ aOR: adjusted odds ratio, using multivariable normal logistic regression* | | | | |
