# Supplementary material for: Ecological and behavioural risk factors of scrub typhus in central Vietnam: a case-control study
Source: Infect Dis Poverty. 2021 Aug 19;10:110. doi: 10.1186/s40249-021-00893-6 (PMC8374119; doi:10.1186/s40249-021-00893-6)
Supplement: Supplementary file 3 — Additional file 3: Table S3. Risk factors of scrub typhus resulting from case-community control analyses. [file 40249_2021_893_MOESM3_ESM.docx]

Table S3 Risk factors of scrub typhus resulting from case-community control analyses

| **Variables** | **Cases** | **Controls** | **aOR (95%CI)^#^** | **P-value** |
| --- | --- | --- | --- | --- |
|  | **n (%)** | **n (%)** |  |  |
| **Gender (male)** | 27 (61.4%) | 30 (36.6%) | 6.3 (1.1-34.4) | 0.035 |
| **Use of personal protective equipment in the field** | 14 (31.8%) | 43 (52.4%) | 0.2 (0.0-1.1) | 0.065 |
| **Urinating in the forest/near bushes/field** | 25 (56.8%) | 22 (26.8%) | 2.6 (0.7-10.0) | 0.170 |
| **Always observing mice around home** | 21 (47.7%) | 22 (26.8%) | 2.7 (0.7-10.3) | 0.146 |
| **Sitting/laying directly on household floor** | 23 (52.3%) | 21 (25.6%) | 35.3 (3.4-368.8) | 0.003 |
| **Household with poor sanitation/conditions** | 41 (93.2%) | 59 (72.0%) | 9.7 (1.2-80.9) | 0.035 |
| **Workplace environment with risk** | 30 (68.2%) | 42 (51.2%) | 3.3 (0.8-12.7) | 0.086 |
| ** The model adjusted for: sex, field work group, use of personal protective equipment in the field, urinating in the forest/near bushes/field, using the same work clothes the next day, changing clothes when at home, always observing mice around home, raising cattle, seeing chickens that you raise have mites, passing riverside, sitting/laying directly on household floor, household with poor sanitation/conditions, household surroundings with risk, workplace environment with risk.* | | | | |
| *^#^ aOR: adjusted odds ratio, using multivariable conditional logistic regression* | | | | |
