# Supplementary figures and images for: Ecological and behavioural risk factors of scrub typhus in central Vietnam: a case-control study
Source: Infect Dis Poverty. 2021 Aug 19;10:110. doi: 10.1186/s40249-021-00893-6 (PMC8374119; doi:10.1186/s40249-021-00893-6)

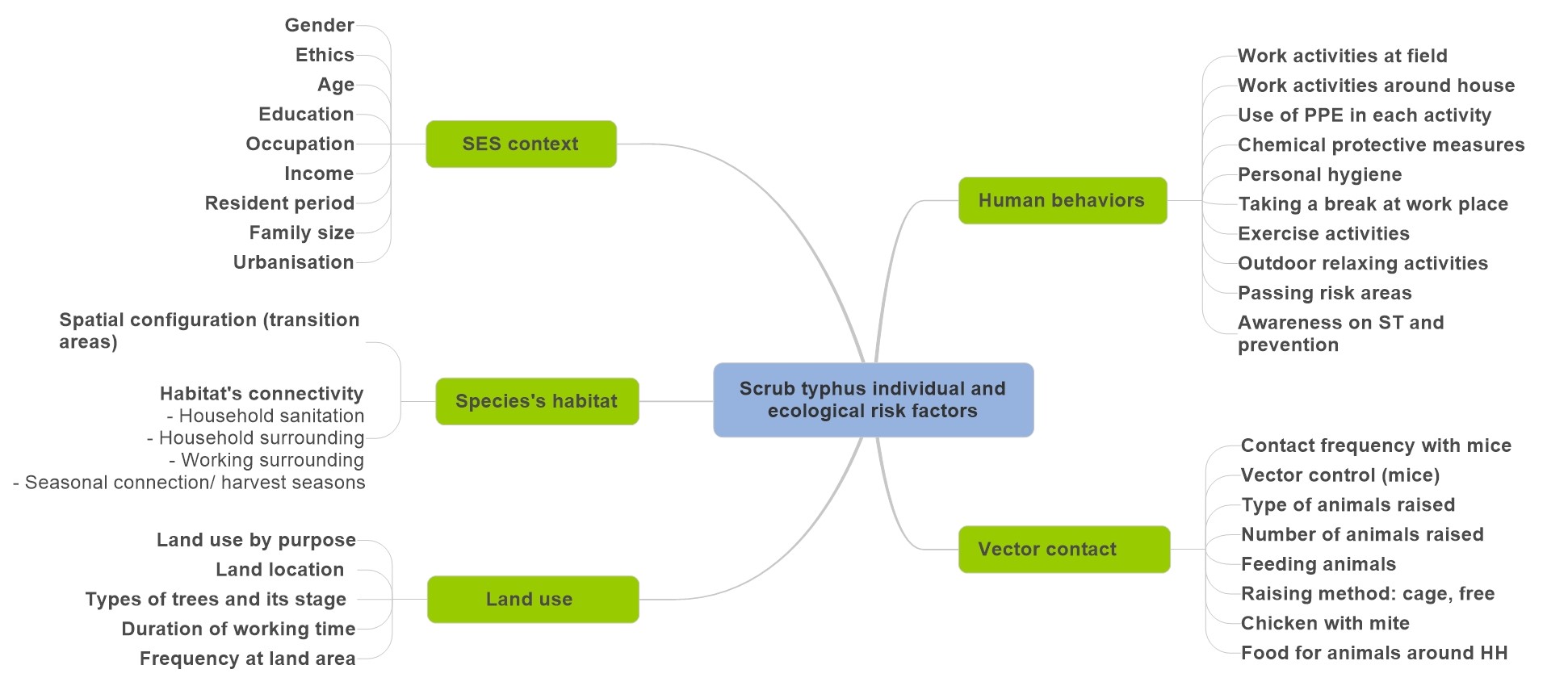

Supplement: Supplementary file 4 — Additional file 4: Figure S1. Visualisation of 5 main items regarding ST risk factors to be assessed in Khanh Hoa. [file 40249_2021_893_MOESM4_ESM.jpeg]
